# Experiment and simulation of novel liquid crystal plasma mirrors for high contrast, intense laser pulses

**DOI:** 10.1038/srep32041

**Published:** 2016-08-25

**Authors:** P. L. Poole, A. Krygier, G. E. Cochran, P. S. Foster, G. G. Scott, L. A. Wilson, J. Bailey, N. Bourgeois, C. Hernandez-Gomez, D. Neely, P. P. Rajeev, R. R. Freeman, D. W. Schumacher

**Affiliations:** 1 The Ohio State University, Columbus, Ohio 43210, USA; 2 Institut de Minéralogie et de Physique des Milieux Condensés, UMR CNRS 7590, Université Pierre et Marie Curie, 75005 Paris, France; 3 Central Laser Facility, STFC Rutherford Appleton Laboratory, Oxfordshire OX11 0QX, UK

## Abstract

We describe the first demonstration of plasma mirrors made using freely suspended, ultra-thin films formed dynamically and *in*-*situ*. We also present novel particle-in-cell simulations that for the first time incorporate multiphoton ionization and dielectric models that are necessary for describing plasma mirrors. Dielectric plasma mirrors are a crucial component for high intensity laser applications such as ion acceleration and solid target high harmonic generation because they greatly improve pulse contrast. We use the liquid crystal 8CB and introduce an innovative dynamic film formation device that can tune the film thickness so that it acts as its own antireflection coating. Films can be formed at a prolonged, high repetition rate without the need for subsequent realignment. High intensity reflectance above 75% and low-field reflectance below 0.2% are demonstrated, as well as initial ion acceleration experimental results that demonstrate increased ion energy and yield on shots cleaned with these plasma mirrors.

Laser facilities with intensities and peak powers exceeding 10^20^ W/cm^2^ and 1 *PW* are now available at laboratories around the world, with several new facilities under construction that will reach 10^22^ W/cm^2^ and 10 *PW*. These intensities exceed the ionization thresholds of all materials (~10^13^ W/cm^2^) by many orders of magnitude and therefore place strict requirements on laser intensity contrast for performing well-controlled studies.

Ultra-intense pulse generation and amplification naturally produces several forms of pre-pulses ranging from *ns* to *fs* timescales prior to the main pulse. Pre-pulse light can originate as amplified spontaneous emission from high gain amplifiers, scattered reflections in multipass amplifiers or retro-reflecting optical paths, imperfect phase correction in grating compressors, and as a ramp up to peak intensity due to laser architecture. Though this light may be significantly less intense than the main pulse–10^−10^ contrast or better is possible in systems designed with pulse cleaning mechanisms–pre-plasma formation sufficient to influence experimental results is commonplace.

Minimizing pre-plasma is critically important for solid target experiments in many cases as hydrodynamic expansion ruins the initially sharp interface. Pre-plasma formation and related target expansion is known to impact hot electron generation^1^ and the ability to control electron acceleration using recently demonstrated structured targets^2^,^3^ as well as the optimization of sub-*μm* ion acceleration^4^,^5^,^6^ and high-harmonic generation^7^.

Several techniques have been developed to achieve this, often requiring significant laser architecture overhaul making them difficult to implement on an existing facility. These include using low-gain optical parametric amplification in the laser front-end^8^, using a third order process like cross polarized wave generation (XPW)^9^ for pre-pulse reduction before amplification, or the installation of fast Pockels cells and spatial filters to reduce early and incoherent pre-pulses^10^.

A simple and commonly used approach is the plasma mirror, typically an anti-reflection (AR) coated fused silica substrate^11^,^12^,^13^,^14^,^15^,^16^,^17^,^18^. The AR coating is critical for good pre-pulse rejection with the resulting low-field reflectance determining the maximum achievable contrast enhancement. A properly focused pulse will then generate a highly reflective plasma surface with its main peak leading edge, creating an ultra-fast temporal filter that enhances intensity contrast by roughly two orders of magnitude at the cost of ~25% of the incident energy. However, each shot damages the affected region so the plasma mirror must be rastered to a clean area or replaced; the requisite alignment and cost remain obstacles to prolonged or high repetition rate pulse cleaning despite much progress on plasma mirror technique^12^,^18^,^19^. A possible alternative is the liquid jet plasma mirror^17^, but it has not yet demonstrated appropriate low-field reflectance due to the lack of an AR coating. Also, such jets currently have the downside of producing a high vapor pressure that will degrade a high intensity short pulse.

We present first results demonstrating freely suspended liquid crystal films as plasma mirrors for pulse contrast enhancement. Previously demonstrated for ion acceleration^20^, the thickness control available with liquid crystal films enables tuning to the etalon minimum thickness, effectively making a self-AR coating (<0.2% reflectance) in the low-field limit. At high intensity we observe characteristic plasma mirror behavior including high quality, >75% reflectance. In this way liquid crystals enable a contrast enhancement of >350, and crucially can do so at high repetition rates with negligible cost. We also introduce a particle-in-cell (PIC) simulation that implements the ionization and collision models necessary to capture dielectric plasma mirror operation for the first time.

## Liquid crystal films as plasma mirrors

Manually formed liquid crystal films for ultraintense laser largets were recently demonstrated using 4-octyl-4’-cyanobiphenyl (8CB)^20^. Film thicknesses were achieved ranging from 10 *nm* to several 10 s of *μm* by controlling film formation parameters such as temperature, applied volume, and wiper speed. This high degree of control was possible due to the molecular layering inherent to the smectic phase, present at room temperature for 8CB. 8CB is generally well-suited for use in high intensity laser experiments as its vapor pressure is below 10^−6^ *Torr* and films formed with it have high structural stability. For this work, liquid crystal films were formed on demand in vacuum using a device we will refer to as the Linear Slide Target Inserter (LSTI), which operates by sliding a Teflon-coated copper wiper over a copper frame aperture. A small volume of liquid crystal, typically 10 *μL* or less, is applied between the wiper and frame prior to operation. Moving the wiper up over the aperture introduces liquid crystal to this area, then pulling the wiper down forms a film in its wake. The LSTI can form films more reliably and over a greater thickness range than achieved by hand, but the polish of the mating surfaces is critical. Since each film contains only 10–100 *nL* of liquid crystal, several hundred films can be formed at low cost from one volume charge of 8CB. Longer operation can be accommodated by a simple syringe apparatus installed within the vacuum chamber. The intrinsic surface tension of the smectic phase produces an optically smooth film surface with extremely uniform film thickness, constant to within our measurement resolution of 2 *nm*. Once aligned to the laser focus, subsequent films form with high position repeatability when using a beveled aperture: less than 2 *μm* RMS variation is possible, well within the Rayleigh range (~10 *μm*) of typical ultra-intense laser experimental setups with solid targets. Although these liquid crystal film techniques were originally developed for targetry, this combination of thickness control, positioning repeatability, low cost, high surface quality, and scalability to high repetition rate formation makes liquid crystal films ideal for use as plasma mirrors.

Liquid crystal films can be optimized for plasma mirror use by selecting the thickness that minimizes low-field reflectance for a given laser wavelength and incidence angle. The low-field reflectance of a thin film etalon is


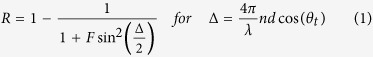


where *F* is the Fresnel reflection coefficient, *λ* is the laser wavelength, *n* is the index of refraction, *d* is the film thickness, and *θ*_*t*_ is the light angle after first surface transmission. 8CB molecules are uniaxial with the crystal axis pointing along the film normal, so *S* and *P* polarizations will experience different reflection coefficients and indices of refraction (*n*_*e*_ = 1.73 and *n*_*o*_ = 1.53 for 8CB at 28 °C^21^) and as such require slightly different optimum thicknesses for pre-pulse rejection.

## Results

### Experiment

**Figure 1:**
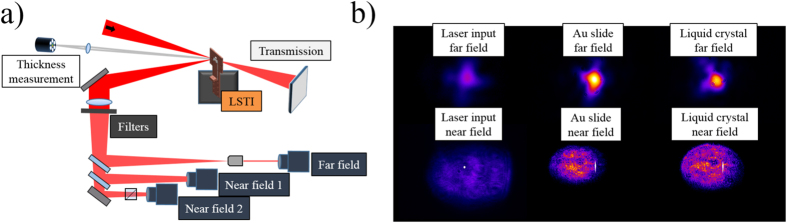
(**a**) Schematic of experimental apparatus and diagnostic layout including a scatter screen for transmitted light observation, and several reflection cameras. The angle of incidence onto the liquid crystal was 15°. (**b**) Near field and far field images of the incident laser, the reflection from an Au-coated glass slide, and from a liquid crystal film, showing high reflection quality. The central dark spot present in the Au and liquid crystal reflected near field images is caused by an aperture in a post-compressor mirror that sends light to laser diagnostics.

A series of shots were taken with *S*-polarized light and relatively low intensity (*I* = 10^11^ *W*/*cm*^2^) to measure the liquid crystal reflectance below the ionization threshold. The vacuum chamber setup with diagnostics can be seen in Fig. 1a. Reflected mode quality is demonstrated in Fig. 1b with far field and near field images. The leftmost “input” images are those from before the final laser grating pulse compressor, the Au slide images provide a quality reference, and the rightmost images are reflections from a liquid crystal film. In general, liquid crystal films are locally flat due to the crystallinity of their molecular structure, but they are affected globally by curvatures induced from aperture edge defects. The laser spot size was tuned for optimal plasma mirror operation at full power resulting in a 0.5 *mm* diameter illuminated region, much smaller than the 4 *mm* film diameter. Finally, the liquid crystal films formed to one uniform thickness across the film diameter because the number of liquid crystal layers could be kept constant.

**Figure 2:**
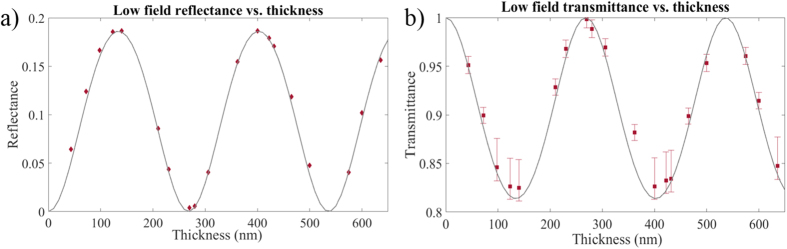
(**a**) Reflectance and (**b**) transmittance data as a function of liquid crystal film thickness for low intensity light. Data points are integrated signals from the cameras, and the curve is a calculation using the etalon reflection from Eq. 1.

Figure 2 shows the low-field reflectance and transmittance as a function of liquid crystal thickness plotted over a curve from Eq. 1; the good agreement with the reflectance and transmittance data indicates good film quality. Error bars (smaller than the data point on the reflectance graph) come primarily from inconsistencies in the camera filtering used on the measurements. For the parameters used here the ideal film thickness is 270 *nm* which corresponds to <0.2% reflected light–this is similar to typical results on ion beam sputter-coated slides and superior to those coated with vapor deposition^22^. Of note is the low reflectance also possible with <30 *nm* films–while tuning to precisely 270 ± 10 *nm* takes several wipes with the current LSTI film formation device, the properties of liquid crystals cause them to preferentially form films of <30 *nm* thickness when the wiper speed is above a certain value. This means that rapid low reflectance films are readily available with the current technology, and more robust pre-pulse reduction can be achieved with longer film thickness adjustments.

**Figure 3:**
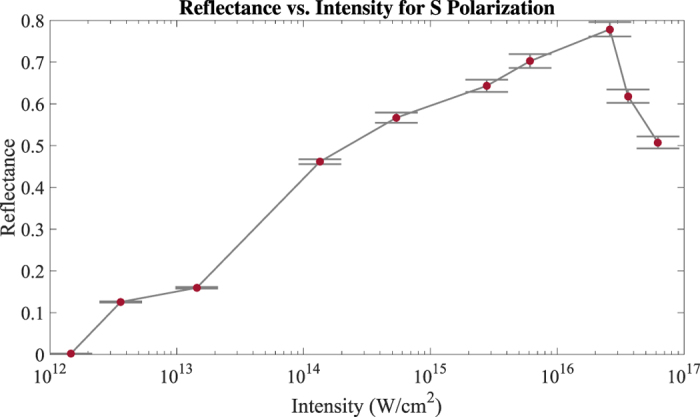
Reflectance vs. intensity showing the onset of plasma reflectance for *S* polarization. As with other materials, a maximum reflectance is achieved near 10^16^ *W*/*cm*^2^.

Reflectance and transmittance of *S* polarized light at a range of intensities is shown in Fig. 3. Here the error bars result from systematic uncertainties in the filters used in the various cameras, each of which was calibrated with spectrophotometry measurements after the experiment. Maximum reflectance occurs near intensities of 1 × 10^16^ *W*/*cm*^2^, similar to other materials^12^,^19^. The *S* polarized reflectance is 75% for the optimum incident intensity, comparable to conventional fused silica plasma mirrors.

A third order cross correlation contrast measurement was not performed due to the moderate laser shot rate. The actual contrast enhancement from a dielectric plasma mirror depends on the specific nature of the laser pre-pulse and the resulting plasma dynamics, however, as is commonly done, we can estimate the enhancement by taking the ratio of the peak high field reflectance to the low field (non-ionizing) reflectance. The resulting factor for a single liquid crystal plasma mirror exceeds 350. Thus, a dual plasma mirror system would in principle yield a contrast enhancement exceeding five orders of magnitude while still retaining >50% transmission of the incident laser energy. Additionally, unlike conventional systems, liquid crystal plasma mirror operation can be continued indefinitely and at low cost using a LSTI or similar device with occasional reservoir resupply.

**Figure 4:**
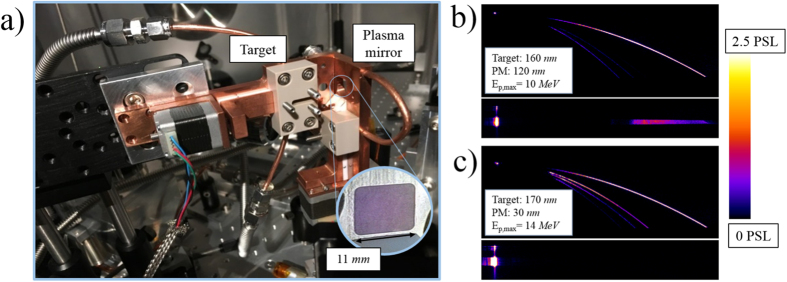
(**a**) Experimental setup including two LSTI devices: one with 11 *mm* aperture (inset) acting as plasma mirror, and other as the target. The distance between the films was 10 *mm*. (**b**) Ion traces from a 160 *nm* thick film after the light was partially cleaned by reflecting from a 120 *nm* plasma mirror film. The curved traces show ions accelerated along the target normal direction measured in a Thomson parabola spectrometer, and the broad line trace is the signal from a magnetic spectrometer placed along the laser axis (45° away). (**c**) The same diagnostics observing ions from nominally the same target thickness but now after superior cleaning from a 30 *nm* plasma mirror, resulting in no laser axis signal and higher maximum proton energy and more ionization among carbon species along the target normal direction. The ion traces have been scaled as indicated in false color so that the signals with lower photostimulated luminescence (PSL) values are visible.

As a practical measure of the effect of the contrast enhancement, two LSTI devices were employed to demonstrate ion acceleration using laser pulse cleaning via liquid crystal plasma mirrors. The experimental setup can be seen in Fig. 4a, which shows one device with an 11 *mm* aperture to serve as a plasma mirror and another with a 4 *mm* aperture as the target. Because the reformable liquid crystal plasma mirrors do not need to be rastered like conventional dielectric mirrors, the plasma mirror LSTI could be placed in the path of the beam reflecting from the final *F*/2 off axis parabola. The device needed to be quite close (10 *mm*) to the target position to have sufficient intensity for plasma generation and reflection at high field. The angle of incidence onto both the plasma mirror and target was 45°.

A Thomson parabola spectrometer^23^ observed target normal directed ions and a magnetic spectrometer recorded ion energies along the laser axis direction. Target thickness could be varied to move between the regimes of various ion acceleration mechanisms while the plasma mirror thickness could be adjusted to change the laser contrast in real time. Figure 4b,c show the target normal and laser axis ion signal from nominally the same target thickness but with two different plasma mirror thicknesses: the 120 *nm* plasma mirror only enhanced laser contrast by a factor of 2, while the 30 *nm* improved it by a factor of 100.

The cleaned shot resulted in higher maximum proton energy–by 40% for the shots shown in Fig. 4–and higher yield for the more ionized carbon states, both indicating a stronger sheath field as is expected from superior contrast. The maximum observed proton dose for the cleaned shots was also higher, by 30% for the data shown here. Low energy laser axis signal was only observed on the poorly cleaned (non-optimal plasma mirror thicknesses) shots–this is indicative of a large spray of ions resulting from target rear surface deformation as a result of prepulse. For the cleaned pulse there is no such expansion and no laser axis ions appear.

### Simulation

Particle-in-cell (PIC) simulations are commonly used to analyze and optimize plasma mirror operation for high harmonic generation from metals^15^,^24^,^25^,^26^ and have played a critical role in the development of that field. PIC simulations have not played an equivalent role in the study of plasma mirror operation for pulse cleaning from dielectric plasma mirrors (DPMs), however, due to the unique challenges involved: several simplifying assumptions typically made to arrive at interpretable results with reasonable computational cost for the former case would invalidate a simulation for the latter. In particular, the initial state of the dielectric substrate, photoionization, and collisions within the resulting plasma play a crucial role.

PIC simulations using metal substrates as targets or plasma mirrors are typically initialized as a singly or multiply ionized plasma to mimic the properties of the conductor and because the substrate is expected to be rapidly heated by the laser pulse. Doing this for a DPM would begin the simulation with the plasma mirror in its “on” (highly reflective) state, completely omitting the pulse cleaning operation. Instead, DPMs must be modeled using neutral particles which can subsequently be photoionized^27^. Further, proper treatment of the low-field response requires modeling the dielectric response of this neutral system. The impinging light must reflect (with appropriate phase shifts), refract, and transmit so that the etalon response is correctly modeled: at the proper thickness for minimum etalon reflection, the multiple reflections from the front and back surfaces of the film must destructively interfere. Next, as the intensity increases, the neutral particles must ionize to produce the plasma that will cause DPM reflection once the density is high enough, approximately at the critical density *n*_*crit*_ = 1.75 × 10^21^/*cm*^3^ for 800 *nm* light. The central, most intense region of the laser beam will reach this threshold first with the spatial wings reflecting later or not at all and this can affect the outgoing spatial mode and so must also be modeled. Sufficient intensity for the spatial mode wings to reach critical density can cause the significantly higher intensity central region to form a highly overdense plasma which will affect the subsequent laser-plasma interaction. Finally, the primary loss mechanism is via absorption in the evolving plasma. Although the transmitted light contains the pre-pulse, the total energy of the pre-pulse is generally quite small. A variety of absorption mechanisms can be at play, such as inverse-Bremsstrahlung or Brunel, varying depending on how long the pulse is and the specific plasma conditions that arise^28^. Collisions within the plasma must be modeled for some of these mechanisms as they cause the electrons to dephase from the driving laser field and to heat the growing plasma. In summary, from before the onset of significant ionization to well above the threshold for turning the DPM on, a simulation must correctly treat five orders of magnitude variation in intensity, or more.

The photoioinization model presents particular challenges in this regime. The appearance intensity when an atom first ionizes can be estimated using the barrier suppression model^29^:


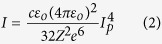


where Z is the ion charge state after ionization, *I*_*p*_ is the ionization potential, *c* is the speed of light, and *e* the fundamental charge. 8CB is mostly hydrogen and carbon with appearance intensities of 1.4 × 10^14^ *W*/*cm*^2^ and 6.4 × 10^13^ *W*/*cm*^2^, respectively. Two limiting cases for possible photoionization mechanisms are multiphoton ionization (MPI) and tunneling ionization. The Keldysh parameter^30^, 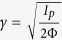 where Φ is the ponderomotive potential, provides a figure-of-merit for identifying which mechanism dominates. Typically it is required that *γ* ≈ 0.5 or lower for tunneling rates to apply^31^. For 800 *nm* light, the appearance intensities given above yield Keldysh parameters of 0.9 and 1.2 for hydrogen and carbon, respectively, suggesting a liquid crystal DPM will operate in the MPI regime. This will, in fact, generally be the case for any dielectric based plasma mirror. Typically intensities up to or above 10^16^ *W*/*cm*^2^ will be present during the operation of a DPM, but ionization of hydrogen and carbon will have saturated well before these intensities are reached such that significant tunneling may never occur, except perhaps for higher ionization stages of carbon. Thus, there will be a significantly overdense plasma near the center of the spot with multiple species present whereas underdense plasma with only first stage ionization contributing will be present farther from the central spot. Tunneling models such as the ADK model^32^ will greatly understate the ionization rates in the MPI regime, but use of MPI models in PIC is uncommon for ultraintense laser simulations.

**Figure 5:**
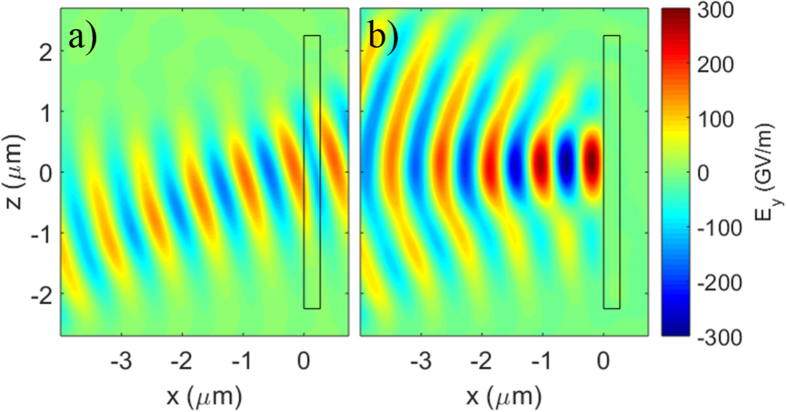
Simulation of target reflection using dielectric and ionization models. The target (black rectangle) thickness was set to 270 *nm* and a dielectric model was used to treat the index of refraction of the liquid crystal 8CB. The laser enters from the left and the laser electric field (color scale) is plotted at the time the peak intensity of 5 × 10^15^ *W*/*cm*^2^ reaches the target. (**a**) With no ionization model, the reflectance is very low. (**b**) The target area is now populated with neutral particles which can ionize, and high plasma reflectance is obtained.

We present PIC simulations of DPMs that, to our knowledge for the first time, address all of the issues described above. This work was done using the code LSP^33^. These simulations begin with relatively cold neutral atoms (1 *eV*) representing 8CB. A dielectric model is employed defining the initial target region on the simulation grid where the atoms reside to have the index of refraction appropriate for 8CB given the input polarization and angle of incidence (*n* = 1.53). For a target thickness of 270 *nm*, the low-field reflectance in the simulation was observed to be 0.2%, close to that found in the experiment, see Fig. 5a. Both tunneling and MPI models were tested, as described below, see Fig. 5b. A collisional plasma was modeled using the Jones algorithm with Spitzer rates capped at 5 × 10^15^ *Hz*^34^. During a simulation, the density can reach several times critical density and temperatures as high as 100 *eV*. The plasma frequency and skin-depth were always resolved, but the extremely short Debye length was not. To avoid the Debye heating instability, an energy-conserving algorithm was employed. To reduce computation time, these simulations were performed in 2D3V, but a 3D correction was applied in post-processing by running many simulations at a range of peak intensities and treating each as a strip of the full 3D problem with the appropriate weights.

**Figure 6:**
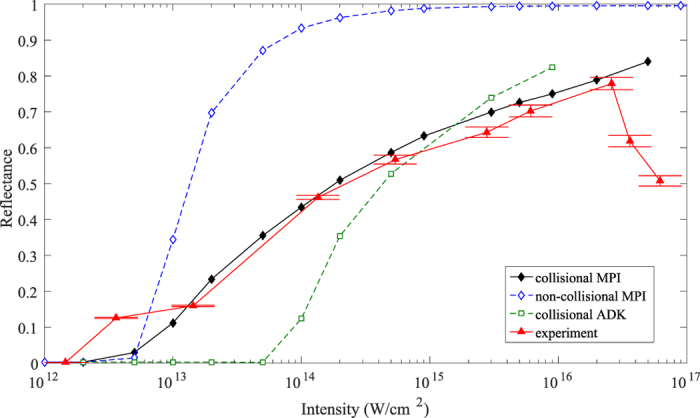
*S* polarization reflectance curves obtained from LSP PIC simulations. An MPI model is required to capture plasma mirror turn-on and a collisional plasma must be modeled to include plasma losses (black compared to green curves). The experimental results are reproduced for comparison (red).

Figure 6 shows the experimental *S* polarization data along with three simulation curves using different models. Collisionality and the choice of ionization model are seen to have a large effect on the simulation results, illustrating the imporantance of these models. The ADK tunneling model (green squares) commonly used in PIC simulations of interactions driven at relativistic intensities drastically underestimates the onset of plasma mirror reflection, as expected. It does better at high intensities, but this is because it eventually saturates the ionization of hydrogen and carbon, even if not at the correct intensities. To our knowledge, there is not a well established MPI model for these conditions. Accordingly, we employed an MPI model^35^ calculating the probability of ionization as 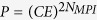 with an adjustable rate coefficient *C* where *E* is the laser electric field and *N*_*MPI*_ is the minimum number of photons required for photoionization. The MPI coefficient of *C* = 1 × 10^−9^ was chosen by matching the reflectance at one intensity only and then using this for the rest of the curve, resulting in an excellent match to experiment (black diamonds vs. red triangles) over three orders of magnitude in intensity. The importance of modeling a collisional plasma can be seen by turning the collisional model off. These results (blue diamonds) overestimate the reflectance by 25% or more, indicating the degree of loss due to excitation of the temporally and spatially varying plasma layer. Without the 3D correction, the simulation results would have been too high by about 5% at the highest intensities and more at lower intensities, indicating 3D effects are also significant. Full 3D simulations will be performed in future work. The lack of roll-off at the highest intensities is interesting and may be due to under-resolving the Debye length, which is computationally difficult.

## Discussion

Presented here are the first experiments demonstrating the utility of freely suspended liquid crystal films as high repetition rate, high quality plasma mirrors for ultra-intense laser experiment and application. The low-field reflectance was measured to be below 0.2%, while the optimum high-field reflectance was above 75%; the resulting contrast enhancement is over two orders of magnitude, comparable to conventional plasma mirrors. The reflected mode is of sufficient quality for these plasma mirrors to be placed either immediately prior to the target laser focus or in a setup with recollimating optics before the target chamber. Initial ion acceleration data proves the utility of the plasma mirrors for efficient ion acceleration from thin targets. The use of liquid crystals permits prolonged, high repetition rate operation compared to conventional AR-coated fused silica substrates. Finally, we have demonstrated novel PIC simulations that are in good quantitative agreement with the measured results and now provide a platform for understanding plasma mirror operation and loss mechanisms when used for contrast enhancement.

In addition to being an effective PM solution, liquid crystal PMs can provide two other benefits beyond conventional PMs. First, the tunable plasma mirror thickness enables the laser contrast to be changed on demand if desired, for example to optimize TNSA using a greater pre-plasma scale length. This functionality is not possible with conventional plasma mirrors and is difficult to replicate with the laser architecture itself, and as such provides an important tool for the study and optimization of processes like ion acceleration. Second, and more importantly for high repetition rate laser operation, using a plasma mirror to turn the beam away from the off-axis parabola typically used for the target focus, just before striking the target, will serve to protect this expensive optic from target interaction debris. Protecting sensitive optics along the laser axis from this sort of damage is already an issue but one that will be exacerbated as higher repetition rate facilities begin operation. Removing the necessity of rastering the plasma mirror by using the reformable LSTI films allows this tight setup to be implemented rather than the typically preferred re-collimating plasma mirror design; the latter requires space and expensive optics and typically serves to degrade the final focus due to elongated beam propagation after reflection from the imperfect plasma mirror surface. A plasma mirror setup using liquid crystal as described can be easily implemented on any existing target chamber and can operate at higher repetition rates and for longer times than currently used devices.

## Methods

The plasma mirror reflectance testing was performed on the Astra laser at the Central Laser Facility, which is a 0.6 *J* 0.1 *Hz* titanium:sapphire based system with 40 *fs* pulse duration. The 100 *mm FWHM* beam was focused by an *F*/7 off axis parabola onto the LSTI-formed liquid crystal film, where the position could be varied to adjust the incident intensity. The laser wavelength was centered on 800 *nm*, and the angle of incidence on to the liquid crystal film was 15°, so the optimum thickness for pre-pulse rejection based on the angle-dependent index of refraction was 270 *nm*.

A ground glass scatter screen was placed behind the LSTI to intercept transmitted light, observed with a 12-bit Andor grayscale CCD camera. Light reflected from the film was recollimated and then sent out of the chamber to several diagnostics: one camera with an objective lens set to observe the near field mode, another watching the near field but through a Wollaston prism to separate polarizations, and a final camera recording the far field image.

Energy could be attenuated coarsely by the insertion of two wedge optics in the laser bay to reduce the intensity by 1000 or finely by a waveplate and polarizer. The reflection and transmission diagnostics were calibrated on shot by a set of far field and near field pickoff cameras watching pre-compressor mirror leakage. Full transmission measurements were taken at various settings with no liquid crystal film present for calibration, while reflection calibration was achieved by mounting a glass slide coated with 200 *nm* of Au onto the LSTI itself and shifting this into the beam using a translation stage.

The ion acceleration results were performed at the Scarlet laser facility, which has a 400 *TW* titanium:sapphire based system. Scarlet fires once per minute with 10 *J* on target in 30 *fs* pulses, and an *F*/2 off-axis parabola that results in a 2 *μm* FWHM focal spot^10^.

For the PIC simulations, the 270 *nm* × 4.5 *μm* plasma mirror was composed of the liquid crystal 8CB raw constituents initialized to a temperature of 1 *eV*: hydrogen and carbon neutral atoms in a ratio of 25:21 (densities *ρ*_*H*_ = 5.35 × 10^22^/*cm*^3^, *ρ*_*C*_ = 4.33 × 10^22^/*cm*^3^). Particles were modeled with a cloud-in-cell particle shape and 10 particles per species per cell, at a spatial resolution of 2.5 *nm* × 3 *nm* with a time step of 0.75 times the Courant limit. The target was preceded by a 4 *μm* vacuum gap. Absorbing boundaries for fields and particles were used on all grid boundaries. The simulations were performed with an explicit field solver and particle advance, as well as energy-conserving force interpolation. For these parameters, the skin depth is well resolved but the Debye length is not for part of the simulation due to the eventual high plasma density and relatively low temperature.

The laser in the simulations was an 800 *nm* Gaussian pulse (60 *fs*, 1.2 *μm*, both defined by the intensity *FWHM*), polarized in the Y direction (equivalent to the *S* polarization of the experiment), entering the grid at a 16° angle of incidence with respect to the target normal, with a peak intensity varied between 2 × 10^12^ *W*/*cm*^2^ and 5 × 10^16^ *W*/*cm*^2^. The target region was overlaid with an *n* = 1.53 block to properly model the thin film reflection characteristics. The reflected light was collected in a plane measuring field flux at the laser outlet boundary. A 3D approximation was achieved by treating each 2D3V simulation as a slice of the 3D pulse profile and several such runs were summed over, weighted by their fractional contribution to the total pulse energy. This took into account the shape of the beam in three dimensions, integrating out to 20 times the beam waist in the full dimension. The equation used to find the 3D reflectance was 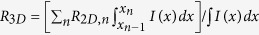, for the 2D simulation reflectivities *R*_2*D*,*n*_ and spatial intensity distributions *I*(*x*).

## Additional Information

**How to cite this article**: Poole, P. L. *et al*. Experiment and simulation of novel liquid crystal plasma mirrors for high contrast, intense laser pulses. *Sci. Rep.* **6**, 32041; doi: 10.1038/srep32041 (2016).

## References

[b1] Ovchinnikov V. M. . Effects of preplasma scale length and laser intensity on the divergence of laser-generated hot electrons. Phys. Rev. Lett. 110, 065007, URL http://link.aps.org/doi/10.1103/PhysRevLett.110.065007 (2013). 23432266 10.1103/PhysRevLett.110.065007

[b2] Jiang S., Krygier A. G., Schumacher D. W., Akli K. U. & Freeman R. R. Effects of front-surface target structures on properties of relativistic laser-plasma electrons. Phys. Rev. E 89, 013106, URL http://link.aps.org/doi/10.1103/PhysRevE.89.013106 (2014). 10.1103/PhysRevE.89.013106 24580345

[b3] Jiang S. . Microengineering laser plasma interactions at relativistic intensities. Phys. Rev. Lett. 116, 085002, URL http://link.aps.org/doi/10.1103/PhysRevLett.116.085002 (2016). 26967419 10.1103/PhysRevLett.116.085002

[b4] McKenna P. . Effects of front surface plasma expansion on proton acceleration in ultraintense laser irradiation of foil targets 26, 591–596, URL http://dx.doi.org/10.1017/S0263034608000657 (2008).

[b5] Wharton K. B. . Effects of nonionizing prepulses in high-intensity laser-solid interactions. Phys. Rev. E 64, 025401, URL http://link.aps.org/doi/10.1103/PhysRevE.64.025401 (2001). 10.1103/PhysRevE.64.025401 11497643

[b6] Schollmeier M. . Laser-to-hot-electron conversion limitations in relativistic laser matter interactions due to multi-picosecond dynamics. Physics of Plasmas 22, URL http://scitation.aip.org/content/aip/journal/pop/22/4/10.1063/1.4918332 (2015).

[b7] von der Linde D. . Generation of high-order harmonics from solid surfaces by intense femtosecond laser pulses. Phys. Rev. A 52, R25–R27, URL http://link.aps.org/doi/10.1103/PhysRevA.52.R25 (1995). 9912328 10.1103/physreva.52.r25

[b8] Shah R. C. . High-temporal contrast using low-gain optical parametric amplification. Opt. Lett. 34, 2273–2275, URL http://ol.osa.org/abstract.cfm?URI=ol-34-15-2273 (2009). 19649068 10.1364/ol.34.002273

[b9] Kourtev S. . Improved nonlinear cross-polarized wave generation in cubic crystals by optimization of the crystal orientation. J. Opt. Soc. Am. B 26, 1269–1275, URL http://josab.osa.org/abstract.cfm?URI=josab-26-7-1269 (2009).

[b10] Poole P. L. . Experimental capabilities of 0.4 pw, 1 shot/min scarlet laser facility for high energy density science. Appl. Opt. 55, 4713–4719, URL http://ao.osa.org/abstract.cfm?URI=ao-55-17-4713 (2016). 27409030 10.1364/AO.55.004713

[b11] Kapteyn H. C., Szoke A., Falcone R. W. & Murnane M. M. Prepulse energy suppression for high-energy ultrashort pulses using self-induced plasma shuttering. Opt. Lett. 16, 490–492, URL http://ol.osa.org/abstract.cfm?URI=ol-16-7-490 (1991). 19773976 10.1364/ol.16.000490

[b12] Ziener C. . Specular reflectivity of plasma mirrors as a function of intensity, pulse duration, and angle of incidence. Journal of Applied Physics 93 (2003).

[b13] Doumy G. . Complete characterization of a plasma mirror for the production of high-contrast ultraintense laser pulses. Phys. Rev. E 69, 026402, URL http://link.aps.org/doi/10.1103/PhysRevE.69.026402 (2004). 10.1103/PhysRevE.69.026402 14995561

[b14] Dromey B., Kar S., Zepf M. & Foster P. The plasma mirror–a subpicosecond optical switch for ultrahigh power lasers. Review of Scientific Instruments 75 (2004).

[b15] Thaury C. . Plasma mirrors for ultrahigh-intensity optics. Nature 3, 424 (2007).

[b16] Cai Y. . Time-resolved measurements on reflectivity of an ultrafast laser-induced plasma mirror. Physics of Plasmas 16, URL http://scitation.aip.org/content/aip/journal/pop/16/10/10.1063/1.3247865 (2009).

[b17] Panasenko D. . Demonstration of a plasma mirror based on a laminar flow water film. Journal of Applied Physics 108, URL http://scitation.aip.org/content/aip/journal/jap/108/4/10.1063/1.3460627 (2010).

[b18] Scott G. G. . Optimization of plasma mirror reflectivity and optical quality using double laser pulses. New Journal of Physics 17, 033027, URL http://stacks.iop.org/1367-2630/17/i=3/a=033027 (2015).

[b19] Pirozhkov A. S. . Diagnostic of laser contrast using target reflectivity. Applied Physics Letters 94, URL http://scitation.aip.org/content/aip/journal/apl/94/24/10.1063/1.3148330 (2009).

[b20] Poole P. L. . Liquid crystal films as on-demand, variable thickness (50–5000 nm) targets for intense lasers. Physics of Plasmas (1994–present) 21, –, URL http://scitation.aip.org/content/aip/journal/pop/21/6/10.1063/1.4885100 (2014).

[b21] Horn R. G. Refractive indices and order parameters of two liquid crystals. J. Phys. France 39 (1977).

[b22] Rödel C. . High repetition rate plasma mirror for temporal contrast enhancement of terawatt femtosecond laser pulses by three orders of magnitude. Applied Physics B: Lasers and Optics 103, 295–302 (2011).

[b23] Morrison J. T., Willis C., Freeman R. R. & Van Woerkom L. Design of and data reduction from compact thomson parabola spectrometers. Review of Scientific Instruments 82, – (2011). URL http://scitation.aip.org/content/aip/journal/rsi/82/3/10.1063/1.3556444. 10.1063/1.3556444 21456736

[b24] Naumova N. M. . Towards efficient generation of attosecond pulses from overdense plasma targets. New Journal of Physics 10, 025022, URL http://stacks.iop.org/1367-2630/10/i=2/a=025022 (2008).

[b25] Thaury C. . Coherent dynamics of plasma mirrors. Nature Physics 4, 631–634, URL http://www.nature.com/nphys/journal/v4/n8/suppinfo/nphys986_S1.html(2008).

[b26] Pukhov A., Baeva T., an der Brügge D. & Münster S. Relativistic high harmonics and (sub-)attosecond pulses: relativistic spikes and relativistic mirror. The European Physical Journal D 55, 407–414, URL http://dx.doi.org/10.1140/epjd/e2009-00187-4 (2009).

[b27] Lawrence-Douglass A. Ionisation effects for laser-plasma interactions by particle-in-cell code. Ph.D. thesis, University of Warwick (2013).

[b28] Gibbon P. Short Pulse Laser Interactions with Matter: An Introduction (Imperial College Press, 2005).

[b29] Delone N. B. & Krainov V. P. Tunneling and barrier-suppression ionization of atoms and ions in a laser radiation field. Physics-Uspekhi 41, 469, URL http://stacks.iop.org/1063-7869/41/i=5/a=R03 (1998).

[b30] Keldysh L. V. Ionization in the field of a strong electromagnetic wave. Soviet Physics JETP 20, 1307 (1965).

[b31] Ilkov F. A., Decker J. E. & Chin S. L. Ionization of atoms in the tunnelling regime with experimental evidence using hg atoms. Journal of Physics B: Atomic, Molecular and Optical Physics 25, 4005, URL http://stacks.iop.org/0953-4075/25/i=19/a=011 (1992).

[b32] Ammosov M. V., Delone N. B. & Krainov V. P. Tunnel ionization of complex atoms and of atomic ions in an alternating electromagnetic field. Soviet Phys. JETP 64, 1191 (1986).

[b33] Welch D. R., Rose D. V., Clark R. E., Genoni T. C. & Hughes T. P. Implementation of a non-iterative implicit electromagnetic field solver for dense plasma simulation. Comp. Phys. Comm. 164, 183–188 (2004).

[b34] Jones M. E., Lemons D. S., Mason R. J., Thomas V. A. & Winske D. A grid-based coulomb collision model for pic codes. Journal of Computational Physics 123, 169–181, URL http://www.sciencedirect.com/science/article/pii/S0021999196900145 (1996).

[b35] Corkum P. B. Plasma perspective on strong field multiphoton ionization. Phys. Rev. Lett. 71 (1993). 10.1103/PhysRevLett.71.1994 10054556

